# Alcohol-Related Brain Damage in Humans

**DOI:** 10.1371/journal.pone.0093586

**Published:** 2014-04-03

**Authors:** Amaia M. Erdozain, Benito Morentin, Lynn Bedford, Emma King, David Tooth, Charlotte Brewer, Declan Wayne, Laura Johnson, Henry K. Gerdes, Peter Wigmore, Luis F. Callado, Wayne G. Carter

**Affiliations:** 1 School of Medicine, University of Nottingham, Royal Derby Hospital Centre, Derby, United Kingdom; 2 Department of Pharmacology, University of the Basque Country, and Centro de Investigación Biomédica en Red de Salud Mental, Spain; 3 Section of Forensic Pathology, Basque Institute of Legal Medicine, Bilbao, Spain; 4 School of Life Sciences, University of Nottingham, Queen’s Medical Centre, Nottingham, United Kingdom; Universidade Federal do Rio de Janeiro, Brazil

## Abstract

Chronic excessive alcohol intoxications evoke cumulative damage to tissues and organs. We examined prefrontal cortex (Brodmann’s area (BA) 9) from 20 human alcoholics and 20 age, gender, and *postmortem* delay matched control subjects. H & E staining and light microscopy of prefrontal cortex tissue revealed a reduction in the levels of cytoskeleton surrounding the nuclei of cortical and subcortical neurons, and a disruption of subcortical neuron patterning in alcoholic subjects. BA 9 tissue homogenisation and one dimensional polyacrylamide gel electrophoresis (PAGE) proteomics of cytosolic proteins identified dramatic reductions in the protein levels of spectrin β II, and α- and β-tubulins in alcoholics, and these were validated and quantitated by Western blotting. We detected a significant increase in α-tubulin acetylation in alcoholics, a non-significant increase in isoaspartate protein damage, but a significant increase in protein isoaspartyl methyltransferase protein levels, the enzyme that triggers isoaspartate damage repair *in vivo*. There was also a significant reduction in proteasome activity in alcoholics. One dimensional PAGE of membrane-enriched fractions detected a reduction in β-spectrin protein levels, and a significant increase in transmembranous α3 (catalytic) subunit of the Na^+^,K^+^-ATPase in alcoholic subjects. However, control subjects retained stable oligomeric forms of α-subunit that were diminished in alcoholics. In alcoholics, significant loss of cytosolic α- and β-tubulins were also seen in caudate nucleus, hippocampus and cerebellum, but to different levels, indicative of brain regional susceptibility to alcohol-related damage. Collectively, these protein changes provide a molecular basis for some of the neuronal and behavioural abnormalities attributed to alcoholics.

## Introduction

Chronic excessive alcohol consumption is a global healthcare problem of epidemic proportion [1]. Alcoholics experience a number of cognitive deficiencies such as learning and memory deficits, impairment of decision making, and problems with motor skills, as well as suffering behavioural changes that include anxiety and depression [2], [3]. These debilitating morbidities arise from the cumulative effects of intoxication and alcohol withdrawal, and may be further exacerbated by nutritional deficiencies such as Wernicke-Korsakoff disorders [2]–[6].

Excessive alcohol consumption can result in a reduction of brain weight, with regional brain atrophy [2], [4], [5]. The production of toxic ethanol metabolites, and their post-translational modification (PTM) and damage of cellular proteins is one of the proposed mechanisms that contribute to neuronal damage [5], [7]–[9].

The first toxic metabolite of ethanol, acetaldehyde, can form adducts with the ε-amino group of lysine residues, and it has been suggested that these covalent modifications can disrupt the function of proteins resulting in cellular injury [10]. One of the major targets of acetaldehyde-mediated adduction is α-tubulin [11], and site specific α-tubulin acetylation can influence microtubule polymerisation [10], [11]. Acetaldehyde-protein adducts have been detected within the white matter, frontal cortex, midbrain, dentate gyrus, and cerebellum in ethanol-fed rats, and in the frontal cortex and midbrain of an alcoholic [7]–[9]. Furthermore, alcohol metabolism may also trigger the production of detrimental protein adducts from products of lipid peroxidation, including 4-hydroxy-2-nonenal (4-HNE), that can bind tubulins and trigger loss of microtubular structures via an increase in their insolubility [12]–[14].

In liver, ethanol consumption also increases protein damage as isoaspartate via the impact of ethanol on the methionine metabolic pathway [15], [16]. Ethanol consumption triggers a reduction in the levels of the methyl donor *S*-adenosylmethionine (SAM), and an increase in the levels of the metabolite *S*-adenosylhomocysteine (SAH). This reduced SAM:SAH ratio inhibits the activity of many methyltransferases including protein isoaspartyl methyltransferase (PIMT), an enzyme that functions to trigger the repair of isoaspartate damaged proteins [17]–[19]. However, it has yet to be determined whether a similar mechanism of ethanol-induced inhibition of PIMT and elevation of isoaspartate damage exists in brain tissue.

Regional brain alcohol-induced pathology may impact upon motor-neuron function, and also influence cognitive behaviour. In an attempt to assess protein damage within a brain region involved in cognitive and social behaviour, we first examined *postmortem* brain tissue from the prefrontal cortex (region BA 9) from 20 human alcoholics and 20 age, gender, and *postmortem* delay matched control subjects. We compared control and alcoholic neuronal tissue histology, and then employed protein profiling to identify prominent neuronal tissue protein changes. An identification of the major brain protein changes provided an insight into structural damage in alcoholic’s brains, for which functional deficits were extrapolated.

## Materials and Methods

### Human Brain Samples and Ethics Statement

The human *postmortem* samples used in this study belong to the brain collection of the Neuropsychopharmacology Research Group at the Department of Pharmacology of the University of the Basque Country (UPV/EHU). (http://www.farmacologia.ehu.es/s0026-home/en/contenidos/informacion/s0026_presentacion/en_farm/presentacion.html).

Brain collection is registered in the National Biobank Register of the Spanish Health Department with the number C.0000035 (https://biobancos.isciii.es/ListadoColecciones.aspx). Human brains were obtained at autopsy from 20 alcoholic and 20 control subjects in the Basque Institute of Legal Medicine, Bilbao, Spain. Brain collection was developed in compliance with policies of research and ethical review boards for *postmortem* brain studies (Basque Institute of Legal Medicine, Bilbao). Spanish legislation at the time of sample collection did not require written informed consent from the next of kin for use of these *postmortem* samples in research. Furthermore, the analysis of *postmortem* brain specimens is not defined as human research by the United States Department of Health and Health Services (DHHS) and Food and Drug Administration (FDA) regulations.

The diagnosis of alcoholism was carried out according to the Diagnostic and Statistical Manual of Mental Disorders (DSM-III-R, DSM-IV or DSM-IV-TR; American Psychiatric Association) or International Classification of Diseases criteria (ICD-10; World Health Organization). All diagnoses were established by clinicians in charge of the patients prior to death. This group included 20 alcoholic subjects with no other diagnosed psychiatric disease. Each alcoholic case was carefully matched for gender, age and *postmortem* delay with a control subject who died by sudden and violent cause with no *antemortem* history of any neurologic or psychiatric disorder. Blood toxicological screening for alcohol and psychotropic drugs was undertaken for each study participant. Tables 1 and 2 summarise the demographic characteristics and the drug history of the subjects included in the microscopy and biochemical studies respectively. Samples from the BA 9 region were macroscopically dissected at the time of autopsy and processed according to the type of study. Brain samples from 10 case-control matched pairs were processed for microscopy, and brain samples from another 10 matched case-control pairs immediately frozen and stored at −80°C until required for biochemical studies. For 5 pairs of BA9 samples used for biochemical studies, additional participant brain tissue from the caudate nucleus, hippocampus, and cerebellum were also obtained.

**Table 1:** Demographic characteristics, *postmortem* delay (PMD), cause of death, and blood toxicological screening of the control (C) and alcoholic (A) subjects analysed by light microscopy.

Case	Gender *(F/M)*	Age *(years)*	PMD *(hours)*	Cause of death	Ethanol in blood *(mg/ml)*	Other drugs in blood
C1	M	54	23	Fall	0	Flurazepam
C2	M	46	16	Heart failure	0	(−)
C3	F	72	12	Heart failure	0	Flecainidine
C4	F	52	20	Heart failure	0	(−)
C5	M	46	8	Aortic dissection	0	(−)
C6	F	38	18	Heart failure	0	Ibuprofen
C7	F	55	22	Fall	0	Anfetamine, nordiazepam
C8	M	59	20	Heart failure	0	(−)
C9	M	57	20	Heart failure	0	(−)
C10	M	46	16	Aortic dissection	0	zolpidem
Mean	4F, 6M	52±3	17±2			
A1	M	68	15	Acute heart and respiratory failure	0	(−)
A2	M	49	10	Acute heart and respiratory failure	1.14	(−)
A3	F	71	16	Acute heart and respiratory failure	0	(−)
A4	F	54	7	Acute heart and respiratory failure	3.04	(−)
A5	M	60	6	Acute heart and respiratory failure	0	Paracetamol
A6	M	46	11	Hemorrhage	0	(−)
A7	F	44	11	Acute heart and respiratory failure	0	(−)
A8	F	46	19	Acute heart and respiratory failure	1.43	(−)
A9	M	56	26	Acute heart and respiratory failure	0	(−)
A10	M	57	17	Acute heart and respiratory failure	0	(−)
Mean	4F, 6M	55±3	14±2			

**Table 2:** Demographic characteristics, *postmortem* delay (PMD), cause of death, and blood toxicological screening of the control (C) and alcoholic (A) subjects used for biochemical studies.

Case	Gender*(F/M)*	Age *(years)*	PMD *(hours)*	Cause of death	Ethanol in blood *(mg/ml)*	Other drugs in blood
C1*	F	66	15	Traffic accident	0	(−)
C2*	M	71	19	Acute heart and respiratory failure	0	Nordiazepam
C3*	M	48	7	Traffic accident	0	(−)
C4*	M	40	18	Fall	0.56	(−)
C5*	F	36	9	Heart failure	0	(−)
C6*	M	66	50	Traffic accident	0	(−)
C7*	M	37	21	Cranoencephalic trauma	0	(−)
C8*	M	54	23	Fall	0	Flurazepam
C9*	M	42	27	Traffic accident	0	(−)
C10*	M	47	26	Work accident	0	Diazepam
Mean	2F, 8M	51±4	22±4			
A1*	F	51	8	Acute heart and respiratory failure	2.98	Nordiazepam
A2*	M	68	15	Acute heart and respiratory failure	0	(−)
A3*	M	50	24	Acute heart and respiratory failure	0	(−)
A4*	M	43	4	Acute heart and respiratory failure	1.64	Chlormethiazole, Metamizole
A5*	F	43	35	Hemorrhage	0	Metamizole, Fluoxetine
A6*	M	71	17	Traffic accident	0.35	(−)
A7*	M	39	19	Hemorrhage	0.44	(−)
A8*	M	53	12	Suffocation	0	(−)
A9*	M	42	20	Hemorrhage	0	(−)
A10*	M	46	16	Suffocation	0.97	(−)
Mean	2F, 8M	51±3	17±3			

The * indicates the subjects for which caudate nucleus, hippocampus, and cerebellum samples were obtained in addition to the prefrontal cortex.

### Brain Tissue Sectioning and Microscopy

**Figure 1:**
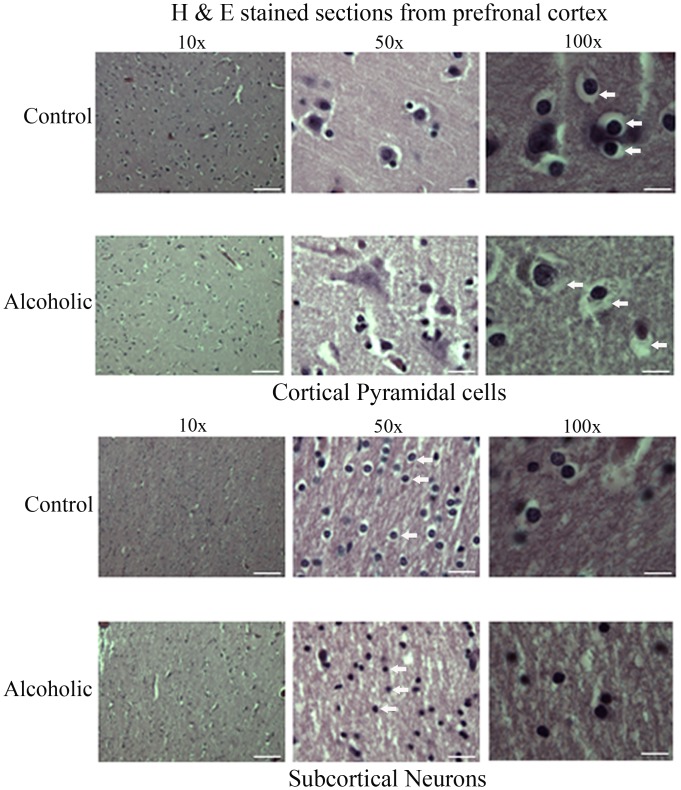
Histological examination of prefrontal cortex neuronal cells from controls and alcoholics. Cortical pyramidal cells (upper two panels), and subcortical neurons (lower two panels) of control and alcoholic tissue sections were visualised by light microscopy. White bar represents 50 μm for 10x, 10 μm for 50x, and 5 μm for 100x lenses. Examples of the differences in the cytoplasm surrounding the nuclei of cells from alcoholic or control tissue sections have been marked with white arrows.

Brain tissues were cryosectioned to 8 μm thicknesses using a Microm HM 550 cryostat, and then mounted onto glass slides and stained with H & E. Brain tissue sections were imaged using a Leica DM4000B light microscope fitted with a 100x/1.25NA, 50x/0.9NA, 20x/0.4NA or 10x/0.25NA N Plan objective lens. Images were captured by a MicroPublisher 3.3RTV camera (QImaging) controlled by OpenLab software (Improvision/PerkinElmer). Regional image capture and analysis were undertaken for all control and alcoholic samples, for which representative images from a control and alcoholic matched pair are included as Figure 1.

### Brain Tissue Homogenisation

Brain tissue samples (∼100 mg) were homogenised at 4°C in 20 volumes of phosphate buffer (200 mM Na_2_HPO_4_, 40 mM NaH_2_PO_4_, 1 mM EDTA, pH 7.4) containing protease inhibitor cocktail (Sigma), using an Ultra-Turrax T8 homogeniser (IKA Labortechnik, Germany). Homogenates were centrifuged at 500×g for 10 min at 4°C in a benchtop centrifuge to pellet the nuclear fraction. The supernatant was then centrifuged at 21,300×g for 40 min at 4°C to produce a crude cytosolic preparation. The pellet from this centrifugation, corresponding to the plasma membrane enriched fraction, was resuspended in 10 volumes of the homogenisation buffer. Nuclear, cytosolic, and membrane enriched fractions were flash-frozen and stored at −80°C until required. Protein concentration in cytosolic and membrane fractions was determined by Lowry protein assay (DC, BioRad, UK) using bovine serum albumin (BSA) as a protein standard.

### One Dimensional Polyacrylamide Gel Electrophoresis (1D-PAGE)

Protein homogenate was separated on 10% Bis-Tris NuPAGE Novex pre-cast gels (20 μg/gel lane) run with 2-(N-morpholino)ethanesulfonic acid (MES) running buffer as described previously [17]. Resolved proteins were either stained with colloidal Coomassie blue and then densitometry performed using an Odyssey laser scanner (LI-COR Biosciences, Lincoln, NE), or proteins electroblotted at 80 V for 2 hours to a polyvinylidene difluoride (PVDF) (Millipore) membrane for Western blotting. All protein homogenates were resolved 2–3 times by gel electrophoresis, and differentially expressed proteins excised from gels and identified by mass spectrometry. Similarly, protein extracts were all resolved 2–3 times for Western blotting analysis, with representative figures from protein staining and blotting experiments included as Figures.

### Matrix Assisted Laser-desorption Ionisation-time of Flight (MALDI-TOF) Mass Spectrometry

Differentially stained proteins were excised from gels and analysed by MALDI-TOF mass spectrometry using a Micromass MALDI (Waters, UK) as described previously [20].

### Liquid Chromatography and Tandem Mass Spectrometry (LC MS/MS)

Tryptic peptides from excised protein bands were run on a Waters QTOF2 hybrid quadrupole mass spectrometer incorporating an integrated capillary LC system as described previously [17]. Further details detailing peptides identified by MALDI-TOF mass spectrometry and LC MS/MS are included as Data S1.

*Western (immuno) blotting:* PVDF membranes were stained with Coomassie blue (Safestain, Invitrogen), and then destained with 50% methanol (v/v), 10% acetic acid (v/v) to visualise proteins, and ensure even protein transfer across the blot. Membranes were then washed in phosphate buffered saline (PBS) containing 0.05% (v/v) Tween 20 (PBS-T), blocked for 1 h at room temperature with 5% (w/v) milk protein in wash buffer, and then incubated overnight at 4°C with the primary antibody of interest in blocking buffer. The antibodies used in this study were directed against the following proteins, and at the dilutions specified: goat polyclonal to spectrin β II (Santa Cruz, sc-7468) at 1∶250; rabbit polyclonal to β-tubulin (Santa Cruz, sc-9104) at 1∶250; mouse monoclonal to α-tubulin (Santa Cruz, sc-8035) at 1∶250; mouse monoclonal to acetylated α-tubulin (Santa Cruz, sc-23950) at 1∶250; mouse monoclonal to protein-L-isoaspartate *O*-methyltransferase (Santa Cruz, sc-100977) at 1∶500; rabbit polyclonal to actin (Santa Cruz, sc-1616-R) at 1∶1000; mouse monoclonal to the α1 catalytic subunit of the Na^+^,K^+^-ATPase (Abcam, ab7671) at 1∶500. A mouse monoclonal antibody to glyceraldehyde 3-phosphate dehydrogenase (GAPDH) (Abcam, ab8245) was used at 1∶3000 as a gel loading control for cytosols, and rabbit polyclonal to actin (Santa Cruz, sc-1616-R) at 1∶1000 as a gel loading control for membrane fractions. Blots were washed in PBS-T and then incubated for 1 hour at room temperature with their corresponding horseradish peroxidase conjugated anti-species secondary antibody (polyclonal goat anti-rabbit, goat anti-mouse or rabbit anti-goat immunoglobulins, all purchased from Dako, products P0448, P0447, P0449 respectively) at 1∶1000 dilution in PBS-T. Antibody immunoreactivity was visualised using SuperSignal West Pico Chemiluminescent substrate (Pierce) with the light generated captured on CL-Xposure X-ray film (Pierce), or using a Chemi Doc device (Bio-Rad). Films were densitometrically scanned using a CanoScan LiDE 700F flatbed scanner (Canon), and the levels of proteins quantified using the public domain image processing program ImageJ, developed at the National Institutes of Health. Alternatively, blots were quantified using QuantityOne software intrinsic to the Chemi Doc charge-coupled device. The results are expressed as GAPDH or actin normalised values. The mean signal density for quantitation of control samples was set at 100% of protein signal. Blots probed for multiple antibody analyses were intermittently antibody stripped with stripping buffer (Restore, Thermo Scientific) by shaking at 125 rpm at 50°C for 30 minutes.

### Quantification of Isoaspartate Levels

The level of isoaspartate in control and alcoholic cytosolic extracts was quantified based upon the method of Aswad and Deight [21]. Protein extracts (100–200 μg of protein) were incubated in a final reaction volume of 100 μl, containing 40 mM K-MES, pH 6.2, 20 μM *S*-Adenosyl-L-[^3^H-methyl]methionine (^3^H-SAM) (8250 dpm/pmol, final specific activity), and 2 μM recombinant PIMT (Promega). Methylation reactions were initiated by the addition of PIMT and incubation for 30 minutes at 30°C. Methylation was terminated by the addition of 1 ml of ice-cold 7% (w/v) trichloroacetic acid (TCA), and protein precipitation on ice for 10 minutes. Protein precipitate was retained by centrifugation for 5 minutes in a refrigerated centrifuge (Eppendorf 5415R) at maximum speed (16 100×g). The supernatant containing unincorporated ^3^H-SAM was removed to waste, and then the precipitate washed with 100 μl of 88% (v/v) formic acid. One ml of 7% TCA was added and protein precipitation performed as before. Precipitate was again collected by centrifugation and extraneous unincorporated ^3^H-SAM removed. The pellet was washed as previous with 100 μl of 88% (v/v) formic acid, and then precipitation with 1 ml of 7% TCA repeated. The protein precipitate was then solubilised in 100 μl of 0.5 M NaOH, 1% (v/v) methanol, 5% (v/v) Triton X-100, and transferred to 10 ml of scintillant and counted for radioactivity incorporation using a Packard Tri-Carb counter. Replicate assays were performed for all assay points from which an average value of radiolabel incorporation determined. Radiolabel incorporation into brain extracts in the absence of PIMT addition, due to endogenous methylation was subtracted from assay values. Isoaspartate quantitation was performed on the 10 matched pairs of control and alcoholic brains.

### Proteasome Activity Assay

Control or alcoholic brain tissue was homogenised in a buffer of 20 mM Tris-HCl pH 7.5, 2 mM ATP, 5 mM MgCl_2_, 1 mM DTT at 4°C. The homogenate was centrifuged at 10, 000×g to pellet cellular debris, and the supernatant transferred to a fresh eppendorf. Fifty μg of homogenate was assayed for proteolytic activity against *N*-succinnyl-Leu-Leu-Val-Tyr-7-amido-4-methyl-coumarin by fluorimetry (excitation wavelength 360 nm, emission wavelength 460 nm). Fluorescent assays were performed in duplicate from which an average value was obtained.

### Statistics

Statistical comparison between data means was performed using a Student’s t-test using GraphPad Prism 4 software, with results expressed as means ± SEM. A *p* value of <0.05 was regarded as statistically significant.

## Results

### Histological Examination of Prefrontal Cortex Neuronal Cells from Controls and Alcoholics

To assess the presence of structural changes within BA 9 tissue that may arise from cumulative ethanol intoxications, we examined cells within brain tissue slices from 10 alcoholics and 10 control subjects matched for age, gender and *postmortem* delay – refer to Table 1. Light microscopy of cells within the cortical pyramidal cell layers revealed that cells from alcoholics exhibited disrupted cytoplasm surrounding their nuclei. These differences were particularly evident with a 100x lens – refer to arrows in Figure 1, upper two panels. An examination of subcortical neurons within the white matter of the alcoholic brain tissue slices also highlighted a reduction in the level of organised cytoplasm surrounding the nuclei. Additionally, subcortical neurons from alcoholics lacked the ordered parallel arrangement of cells seen with control subjects. These differences were well distinguished with a 50x lens – refer to arrows in Figure 1, lower two panels.

### Profiling of Cytosolic Proteins from the Prefrontal Cortex of Control and Alcoholic Subjects and Western Blotting

**Figure 2:**
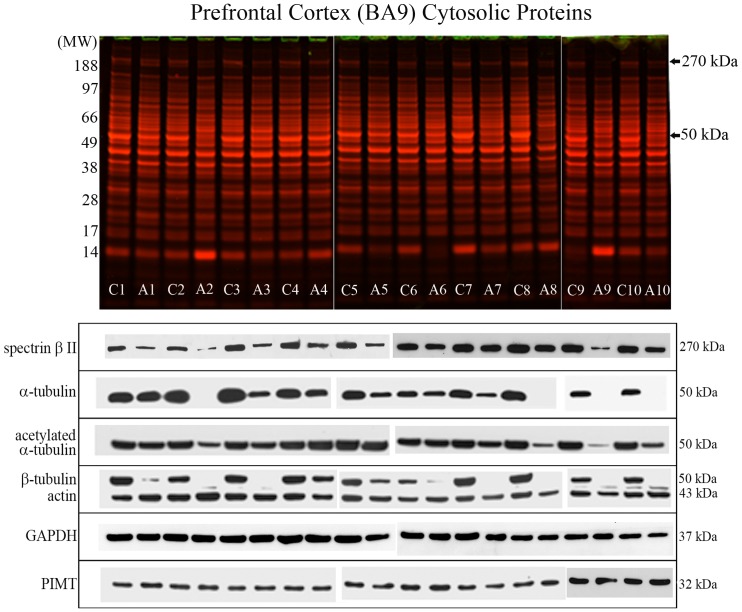
Profiling of cytosolic proteins from the prefrontal cortex of control and alcoholic subjects. Control (C) or alcoholic (A) prefrontal cortex cytosolic proteins were resolved by 1D PAGE and proteins stained with colloidal Coomassie. Alcoholic subjects displayed a prominent reduction in the level of protein staining at protein bands of ∼270 kDa and ∼50 kDa (upper panel). Protein levels were visualized by Western blotting (lower panel). Abbreviations: GAPDH, glyceraldehyde 3-phosphate dehydrogenase; PIMT, protein-L-isoaspartate *O*-methyltransferase.

To investigate further the reduced and disorganised cytoplasm or cytoskeleton observed for alcoholic brain tissue, we homogenised BA 9 tissue from control and alcoholics and examined protein profiles by 1D PAGE. Due to limited sample availability, and to further validate this observation with more subjects, 1D PAGE was performed with an additional population of 10 matched control and alcoholic subjects, the details of which are listed in Table 2. Visual inspection of 1D PAGE protein profiling of cytosolic proteins revealed only two major recurrent protein level differences between control and alcoholic groups. There was a prominent reduction in the level of an ∼270 kDa protein and an ∼50 kDa protein, and these changes were visible in all alcoholics when compared to their control matched counterparts – refer to Figure 2, upper section. Protein bands at these molecular weights from six individuals were removed and proteins identified by mass spectrometry. The ∼270 kDa protein band was identified as spectrin β II, and the ∼50 kDa protein band identified as a mixture of peptides from different forms of α-tubulin and β-tubulin – refer to Table 3 and Data S1.

**Table 3:** Mass spectrometry identification of differentially expressed proteins from the prefrontal cortex of control and alcoholic subjects.

Protein MW(kDa)	Cellularlocalisation	MOWSEscore	Proteinidentification	UniProtKB/SwissProt entry	Protein function
50	Cytosolic	133	α-tubulin	Q71U36§	Microtubule activity, cytoskeleton,protein movement.
50	Cytosolic	159	β-2-tubulin	Q9BVA1†	Microtubule activity, cytoskeleton,protein movement.
270	Cytosolic & membraneassociated	345*	β-spectrin	Q01082	Cytoskeleton, protein movement.
112	Trans-membranous	724*	α3-subunit ofNa^+^,K^+^-ATPase	P13637	Catalysis of ATP coupled to the exchange of sodiumions and potassium ions across the plasma membrane.

§ UniProtKB/SwissProt entry of α-1A-tubulin.

† UniProtKB/SwissProt entry of β-2B-tubulin.

*Values from LC-MS/MS.

**Figure 3:**
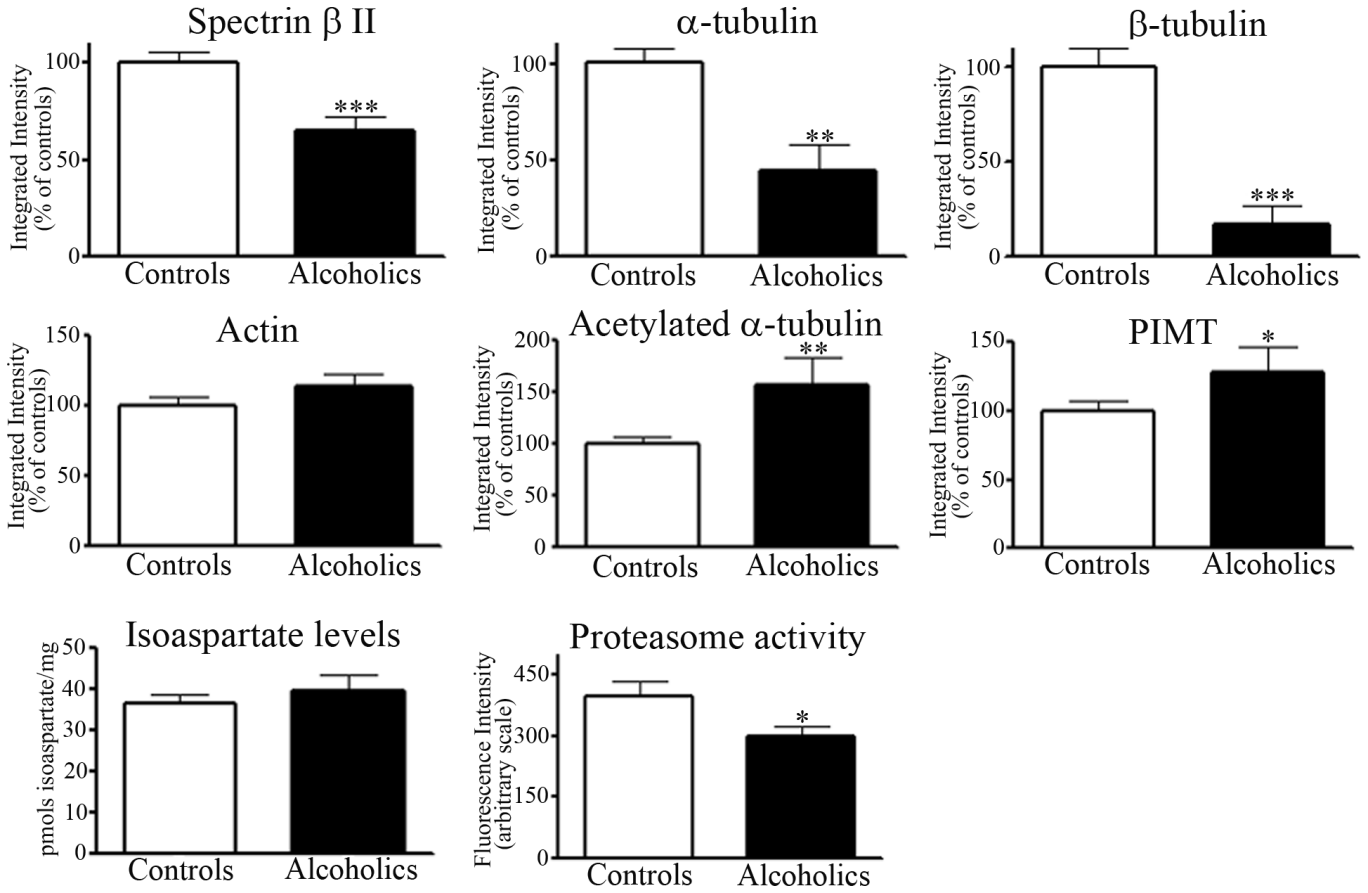
Quantification of protein level and activity differences between control and alcoholic subjects. Protein levels were quantified by Western blotting, and the levels of isoaspartate protein damage and proteasome activity determined. For quantitation of the ratio of acetylated α-tubulin to total α-tubulin, the figure is representative of those subjects that displayed visible total α-tubulin signal (6 of the 10 subjects assayed). For marked significance: * = *p*<0.05, ** = *p*<0.01, *** = *p*<0.001. Abbreviations: PIMT, protein-L-isoaspartate *O*-methyltransferase.

To validate and quantify this dramatic loss of the cytoskeletal proteins β-spectrin, and α- and β- type tubulins specifically within alcoholic brain tissue, we assessed their protein levels by Western blotting – Figure 2 lower section. Western blot analyses confirmed a significant 36% decrease in spectrin β II (*p = *0.0002), significant 56% decrease in α-tubulin (*p = *0.0017) and significant 83% decrease in β-tubulin levels (*p*<0.0001) for the 10 alcoholic subjects – Figure 3. The alcoholic subjects for which the level of either α- or β-tubulins were below the Western blotting detection threshold have been expressed as 100% protein loss. Cytosolic GAPDH levels were stable throughout all control and alcoholic subjects investigated and were used for protein normalisation.

The cellular cytoskeletal network is composed of microtubules, intermediate filaments, and microfilaments. The major protein constituent of microfilaments is actin, hence we also quantified actin protein levels to determine if cytoskeletal loss was extended to actin in microfilaments. Actin levels increased by 16% in alcoholic subjects, but this was not significant – Figures 2 & 3.

### Examination of Protein PTM and Damage in Control and Alcoholic Brain Tissue

We investigated possible PTMs of tubulins that may have contributed to an alteration of their cytosolic level, or that may influence tubulin stability or turnover. We quantified the level of acetylated α-tubulin in all cytosolic protein samples by Western blotting with an antibody that specifically recognizes acetylated Lysine-40– Figure 2. The relative proportion of acetylated α-tubulin to total α-tubulin was quantified as a significant 56% increase (p = 0.0094) for alcoholic subjects when compared to their matched controls – Figure 3.

Secondly, α- and β-tubulins can accumulate isoaspartate damage *in vivo* if PIMT activity is inhibited or removed [17], [22], [23]. We quantified the levels of total isoaspartate protein damage in the cytosolic proteins from control and alcoholic brains, and also the levels of cytosolic PIMT enzyme by Western blotting – Figures 2 & 3. There was a 9% increase in total cytosolic isoaspartate levels within the alcoholic brains but this did not reach significance. In contrast there was a significant 28% increase (*p* = 0.0438) in cytosolic PIMT protein levels in alcoholics. Due to the relatively low total levels of isoaspartate within control or alcoholic brains, an examination of the specific levels of isoaspartate protein damage within α- and β-tubulins was not undertaken.

The proteasome complex is responsible for the turnover of the majority of cytosolic proteins including tubulins [24], [25]. Hence we also quantified proteasomal activity of brain tissue from the 10 control and 10 alcoholic subjects. There was a significant 25% reduction in proteasomal activity in the alcoholic subjects (*p* = 0.0426) – refer to Figure 3.

**Figure 4:**
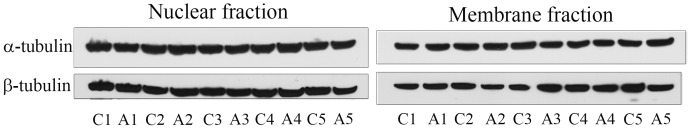
Western blot of α- and β-tubulins in nuclear and membrane fractions from the prefrontal cortex of control and alcoholic subjects. Control (C) or alcoholic (A) prefrontal cortex nuclear and membrane fractions were resolved by 1D PAGE and proteins Western blotted for α- and β-tubulins.

We also examined the levels of α- and β-tubulin within nuclear and membrane fractions to assess whether the reduced cytosolic protein levels seen in alcoholics arose from protein translocation to these other cellular compartments. After 1D PAGE and Western blotting there were no significant changes in the levels of α- or β-tubulins in the nuclear or membrane fractions of alcoholic brain tissue when compared to control subjects – Figure 4. This suggested that α and β-tubulin protein loss in the prefrontal cortex of alcoholics was limited to the cytosol.

### Profiling of Membrane-fraction Proteins from the Prefrontal Cortex of Control and Alcoholic Subjects

**Figure 5:**
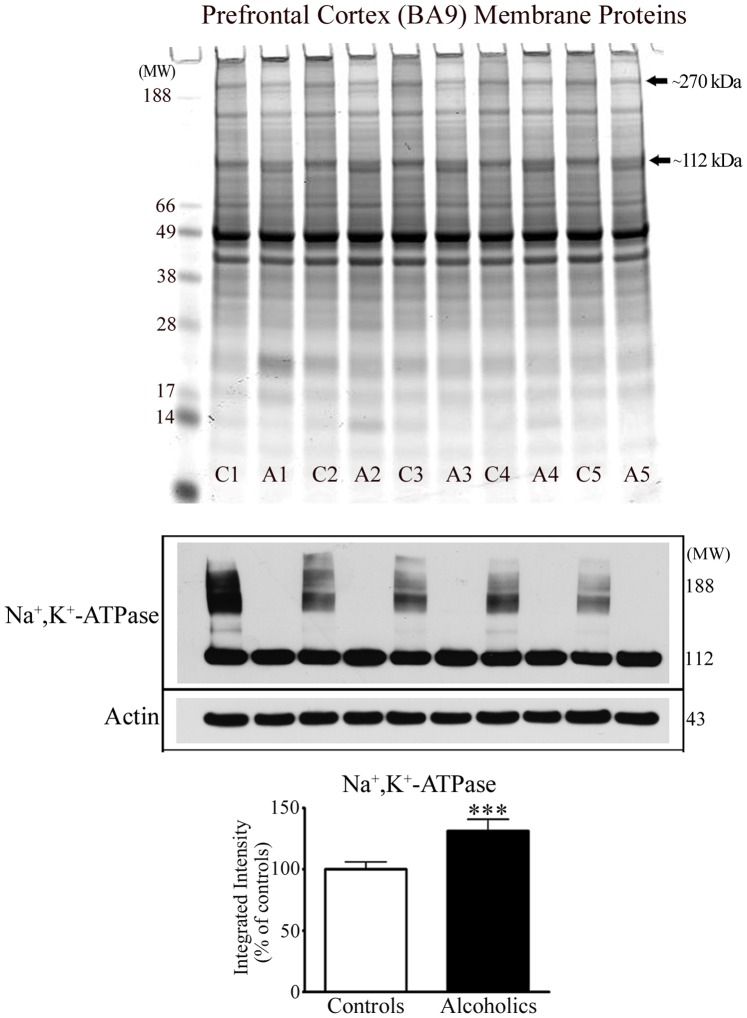
Profiling of membrane-fraction proteins from the prefrontal cortex of control and alcoholic subjects. Control (C) or alcoholic (A) prefrontal cortex membrane fraction proteins were resolved by 1D PAGE and proteins stained with colloidal Coomassie. Alcoholic subjects displayed a prominent reduction in the level of protein staining at a protein band of ∼270 kDa and upregulation at ∼112 kDa (upper panel). Protein levels of transmembranous (∼112 kDa) Na^+^,K^+^-ATPase α-subunit, and oligomeric forms of the α-subunit were visualized by Western blotting (middle panel). The level of transmembranous α-subunit was quantified using actin for normalisation (lower panel). For marked significance: *** = *p*<0.001.

One dimensional PAGE profiling of proteins from the membrane fractions of control and alcoholic subjects resulted in the detection of two visually prominent protein changes. There was a reduction in the protein levels of an ∼270 kDa protein in alcoholic subjects, and an increase in the levels of a protein doublet at ∼112 kDa protein – Figure 5 upper panel. Protein bands at these molecular weights from 5 control and 5 alcoholic subjects were excised from gels and analysed by mass spectrometry. Similar to the cytosolic protein analysis, the reduced ∼270 kDa protein was identified as spectrin β II. Both protein doublet bands at ∼112 kDa protein were identified as the α3 subunit of the Na^+^,K^+^-ATPase – refer to Table 3, and Data S1.

In concurrence with the protein staining data for the Na^+^,K^+^-ATPase α-subunit, Western blotting confirmed a broader immunoreactivity of monomeric, membrane-associated α-subunit in alcoholic subjects; quantified as a combined 31% significant increase (*p = *0.0002). Western blotting also showed that oligomeric forms of α-subunit present in control subjects were virtually absent in alcoholics, suggesting a functional deficit in α-α oligomerisation – Figure 5 middle and lower panels.

### Profiling of Cytosolic Proteins from the Caudate Nucleus, Hippocampus and Cerebellum of Control and Alcoholic Subjects

**Figure 6:**
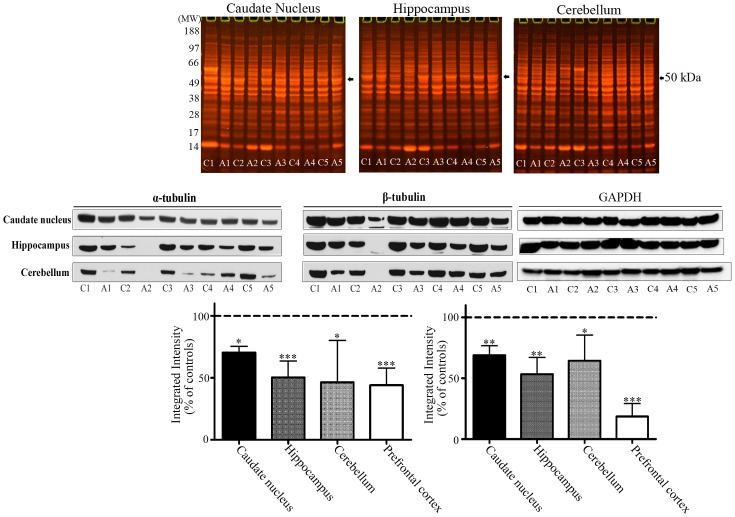
Profiling of cytosolic proteins from the caudate nucleus, hippocampus and cerebellum of control and alcoholic subjects. Control (C) or alcoholic (A) cytosolic proteins from caudate nucleus, hippocampus, and cerebellum were resolved by 1D PAGE and proteins stained with colloidal Coomassie (upper panel). The positions of the ∼50 kDa α- and β-tubulin protein bands are marked with arrows. Cytosolic proteins were Western blotted for α- and β-tubulin, and glyceraldehyde 3-phosphate dehydrogenase (GAPDH) (middle panel), and protein level changes quantified using GAPDH for normalization (lower panel). For marked significance: * = *p*<0.05, ** = *p*<0.01, *** = *p*<0.001.

We wanted to ascertain if loss of α- and β-tubulins in alcoholic brains was a regional phenomenon restricted to the prefrontal cortex. Thus for those brain regions for which we also had access to additional tissues, we examined the levels of cytosolic α- and β-tubulins within caudate nucleus, hippocampus, and cerebellum from 5 matched pairs of control and alcoholic subjects (subject details included in Table 2). Cytosolic proteins from each of the brain regions were resolved by 1D PAGE and Western blotted with the specific anti-α- and anti- β-tubulin antibodies – Figure 6 upper and middle panels.

Quantitation of Western blots showed a significant reduction in the total levels of α- and β-tubulins in all brain regions, but the level of reductions were region specific. There was a significant 30% decrease (*p* = 0.0198) in α-tubulin and a significant 31% decrease (p = 0.0081) in β-tubulin in the caudate nucleus. There was a significant 50% (*p = *0.0004) and 47% (*p = *0.0054) decrease in α- and β-tubulin respectively in the hippocampus, and a significant 54% (*p = *0.0044) and 36% (*p = *0.0486) decrease in α- and β-tubulin in the cerebellum. However, the prefrontal cortex displayed the greatest reductions in α-tubulin (56% decrease, *p = *0.0002) and β-tubulin levels (84%, *p*<0.0001) – Figure 6 lower panels.

## Discussion

An examination of the molecular abnormalities that arise as a consequence of cumulative ethanol intoxications will assist with an understanding of the development of tissue pathology. In addition, a characterisation of ethanol-induced molecular changes may also provide an insight into the molecular adaptations associated with tolerance, dependence, and an alcoholic’s behavioural abnormalities. Collectively, research in this field will provide a basis for targeted therapeutics that counter debilitating morbidity, and lower the incidence of mortality.

We assessed alcohol-related neuronal tissue damage of the prefrontal cortex using light microscopy, and were intrigued to see clear histological distinction between cells of alcoholics and their matched controls. This led us to undertake one dimensional protein profiling of cytosolic proteins from alcoholics and controls and we identified the major protein losses as that from α- and β-tubulins, and spectrin β-chain.

Alpha and β-tubulins localise as heterodimers, and are major components of microtubules. Microtubules are non-covalent cytoskeletal polymers that provide a cellular protein network. Microtubules influence cell shape, motility, and stability, and are central to the functioning of countless cellular processes including mitosis, and vesicular transport [26], [27]. Microtubules exist as both dynamic and stable polymers, with transitions between these states in sub-populations of microtubules influenced by PTMs and microtubule-associated proteins (MAPs) [26], [27]. Hence the loss of functional tubulins seen in the BA 9 tissue of alcoholics would be expected to result in a disruption of the cytoskeletal network, and impinge on the many cellular trafficking processes that are tethered to the cytoskeletal architecture.

Similar to tubulins, spectrins are a family of proteins that are major structural components of the cytoskeleton. Spectrins (as α and β heterodimers) act as molecular scaffolds that associate with the cytoplasmic surface of the plasma membrane, and form a molecular lattice that links the plasma membrane to actin and the microtubule cytoskeleton [28], [29]. Spectrin functions include maintenance of cell shape, and arrangement of transmembranous proteins. Additionally, brain spectrins function in the association of vesicles to the microtubular network, and influence synaptic vesicle stabilization and release [28], [30]. Hence the reduction in spectrin levels seen with alcoholic subjects would also likely disrupt cellular trafficking processes, and the triggering of synaptic neurotransmission events.

Thus collectively, we propose that a reduction of the cytoskeletal architecture provides a rationale for the profound differences in the prefrontal cortex neuronal histology of alcoholics, and likely contributes to the cognitive and learning impairments experienced by alcoholics.

We next considered molecular mechanisms that could explain the selective damage and loss of these cytoskeletal proteins in alcoholics. Ethanol can be converted in the cytosol to acetaldehyde via the action of alcohol dehydrogenase, and in brain this conversion may also be undertaken by catalase and cytochrome P450 enzymes [31]. Acetaldehyde is a reactive compound and readily adducts a number of cytosolic proteins including tubulins [10], [11]. We detected an ∼1.5-fold increase in the ratio of acetylated α-tubulin to total α-tubulin in the alcoholics. Similarly, an increase in the relative proportion of acetylated α-tubulin (to total α-tubulin) within liver or liver cells as a consequence of ethanol consumption or exposure has been reported [32]. In liver, this increased α-tubulin acetylation influences microtubule hyperstabilisation and inertness, with an associated impairment of protein trafficking [32], [33], but at present the influence of ethanol consumption on brain tubulin acetyltransferases or deacetylases, and the functional consequences of increased tubulin acetylation have not been determined.

Another potential source of ethanol-induced damage to α- and β-tubulin could arise through an increase in their protein damage as isoaspartate. We detected a 9% increase in total cytosolic isoaspartate levels within the alcoholic brains, although this did not reach significance. It is still feasible that isoaspartate levels may increase significantly specifically within α- and β-tubulins, but since the total levels of cellular isoaspartate were low, quantitation of individual protein isoaspartate levels was not attempted.

An increase in isoaspartate protein damage in alcoholic subjects could be countered by upregulation of cellular PIMT levels to trigger isoaspartate repair. We detected a significant 28% increase in cytosolic PIMT protein levels in alcoholic tissue. Similarly, a proteomic study of synaptic proteome changes in the superior frontal gyrus (SFG) and occipital cortex (OC) of control and alcoholic *postmortem* tissue reported a significant 30% increase in PIMT protein levels in the SFG and a 50% increase in PIMT protein levels in the OC of alcoholic subjects [34]. The molecular mechanism by which PIMT protein levels are elevated has yet to be determined, and it will be of interest to establish if it reflects a compensatory means to counter an increase in cellular stress and isoaspartate protein damage.

The 26S proteasome complex is the predominant cellular protease that target proteins for degradation after their prior ubiquitination [25]. Additionally, oxidised, modified and/or damaged proteins may also be degraded by 20S proteasomes directly [35], [36]. Hence one possible mechanism to explain the loss of these cytoskeletal proteins is that ethanol induces PTMs and protein damage, and this triggers protein elimination by the proteasome. However, when quantified there was a significant 25% fall in the proteasome activity of alcoholic tissue. Thus although the proteasome may still target clearance of these proteins, its global cellular activity was compromised in the brains of alcoholics.

The reduced levels of cytosolic proteins did not reflect translocation to either the nuclear or membrane fractions. Protein profiling of the membrane fraction also demonstrated that spectrin β II was present at a reduced level in the membrane-enriched fraction from alcoholics. The membrane-associated α-subunit of the Na^+^,K^+^-ATPase was visualised and evidenced as two protein bands of similar molecular weight. Western blotting enabled us to quantify a significant increase in the transmembranous level of the α-subunit of the Na^+^,K^+^-ATPase, which presumably reflected immunoreactivity of both bands of the protein doublet.

The Na^+^,K^+^-ATPase catalyses the hydrolysis of ATP coupled to the asymmetric exchange of intracellular Na^+^ for extracellular K^+^ across the plasma membrane, and participates in generation and maintenance of a membrane potential [37]. Native Na^+^,K^+^-ATPase is heterotrimeric, composed of three subunits: α, β, and γ. The α-subunit is the catalytically active ion transporting subunit. The α1 isoform is ubiquitously distributed, whereas the α3 isoform is expressed in neurons [37], [38].

Western blotting with the Na^+^,K^+^-ATPase α-subunit antibody revealed that stable higher molecular weight (oligomeric) forms of the α-subunit present in controls were absent (or just visible with long Western blot exposures) in alcoholic subjects. The existence of α-α oligomers that are resistant to SDS-PAGE conditions have been documented by independent groups [39], [40].

Ethanol exposure can partially inhibit Na^+^,K^+^-ATPase pump activity in the cerebellum of rats, although the precise mechanism for pump inhibition has yet to be defined [41]. Defective Na^+^,K^+^-ATPase α-subunits result in a rapid-onset dystonia Parkinsonism, and other behavioural and cognitive deficits [38], [42]–[44]. Hence it is provocative to postulate that alterations in α-subunit levels or function and/or oligomerisation could also contribute to some of the behavioural symptoms exhibited by alcoholics.

We also examined tissue from additional brain regions and detected a significant reduction in α- and β-tubulin protein in the caudate nucleus, hippocampus, and cerebellum, but not to the levels of protein loss recorded for the prefrontal cortex. The differential vulnerability of brain regions could reflect varied penetrance across the blood brain barrier of the toxic metabolites of ethanol, such as acetaldehyde and/or their specific regional production.

### Study Limitations

Although we have undertaken a quantitative evaluation of protein levels using Western blotting we appreciate this is not without methodological limitations. Antibody binding to a target protein may be masked or sterically hindered due to the presence of PTMs. This provides a rationale to explain why the antibody directed against acetylated α-tubulin exhibited a higher immunoreactivity signal than the antibody directed against the full-length α-tubulin protein – refer to Figure 2. Hence the total levels of cytoskeletal protein degradation in alcoholic brain tissue are clearly significant (and visibly prominent after 1D-PAGE and protein staining), but levels of protein degradation may possibly be overestimated using immunological reactivity.

Direct comparison with other proteomic studies of alcoholic tissue from the prefrontal cortex is problematic due to differences in alcoholic subject age, cumulative alcohol intake, as well as variability of regional sampling, protein preparation and methodology etc [45]–[47]. Nevertheless, other independent two-dimensional (2D) proteomic studies have also reported reductions in alcoholic’s brains of the protein levels of α- and β-tubulins [45]. Within our study patients differ in many parameters such as blood toxicology at death, but by matching each pair of controls and alcoholics for age, gender, and *postmortem* delay, robust and universal changes in protein levels and modifications were revealed. A more substantive proteomic analysis by 2D separation techniques may reveal additional protein level changes, however, we were primarily concerned with prominent visual differences for which a related function could be postulated; hence a comprehensive 2D-PAGE analysis was considered beyond the scope of this primary publication. Furthermore, 2D proteomic studies utilise protein fractionation and protein precipitation methods to enrich components of the proteome for analyses [34], [45]–[47], and this could mask protein differences visible by primary 1D PAGE screening.

An insight into changes within *postmortem* tissue of the frontal cortex of alcoholic subjects has also been provided using gene arrays [48]–[50]. These studies document numerous changes in gene expression levels that include reduced expression of elements of the ubiquitin-proteasome system in alcoholics [48]–[50], and reduced expression of a beta III spectrin in cirrhotic alcoholics [50]. Thus it remains likely that significant protein level changes evidenced in the prefrontal cortex of alcoholic subjects arise from alteration of transcriptional and translational mechanisms.

In summary, we report profound reductions in the levels of the cytoskeletal proteins α- and β-tubulin, and spectrin β II in alcoholic subjects that correlate with altered neuronal cell organisation and cell patterning visible by light microscopy. The known susceptibility of these proteins to protein damage or regulatory PTMs provides a putative mechanism to explain their targeted loss of protein level and function. Altered protein level and PTMs of the cytoskeletal architecture, and also disruption of the functional activity of the Na^+^,K^+^-ATPase provides a molecular basis for some of the altered neuronal and behavioural manifestations encountered by alcoholics, and may also contribute to the atrophic brain macrostructure that is phenotypic of an alcoholic.

## Supporting Information

Data S1 **MALDI-TOF and LC-MS/MS data for α-tubulin, β-tubulin, spectrin β-chain, and the α3-subunit of Na^+^,K^+^**-**ATPase.**
(DOCX)
