# Supplementary material for: Alcohol-Related Brain Damage in Humans
Source: PLoS One. 2014 Apr 3;9(4):e93586. doi: 10.1371/journal.pone.0093586 (PMC3974765; doi:10.1371/journal.pone.0093586)
Supplement: Data S1 — MALDI-TOF and LC-MS/MS data for α-tubulin, β-tubulin, spectrin β-chain, and the α3-subunit of Na+,K+-ATPase. (DOCX) [file pone.0093586.s001.docx]

**Supplementary Data S1**

**MALDI-TOF mass spectrometry:**

Coomassie stained protein bands of the ~50 kDa protein from control or alcoholic subjects were excised and analysed by MALDI-TOF mass spectrometry. Twelve tryptic peptides that corresponded to approximately 42 % protein coverage of α-1A-tubulin were detected. These peptides generated a MOWSE probability score of 117. The ~50 kDa protein band also liberated fifteen peptides that corresponded to approximately 45 % protein coverage of β-2B-tubulin, and generated a MOWSE probability score of 159. For this MALDI-TOF mass spectrometry analysis a MOWSE score of greater than 66 was considered a significant match (*P* < 0.05) of tryptic peptides to those present within protein databases, to enable confident protein identification.

**Tubulin α-1A-chain peptides:**

**St-End Observed Mr(expt) Mr(calc) Delta Sequence**

85-96 1410.8100 1409.8027 1409.7667 0.0360 R.**QLFHPEQLITGK**.E

97-105 1023.4700 1022.4627 1022.4417 0.0210 K.**EDAANNYAR**.G

113-121 1069.6200 1068.6127 1068.5815 0.0312 K.**EIIDPVLDR**.I

216-229 1718.8800 1717.8727 1717.8747 -0.0020 R.**NLDIERPTYTNLNR**.L

230-243 1487.9000 1486.8927 1486.8719 0.0209 R.**LISQIVSSITASLR**.F

244-264 2409.1900 2408.1827 2408.2012 -0.0185 R.**FDGALNVDLTEFQTNLVPYPR**.I

265-280 1756.9500 1755.9427 1755.9559 -0.0132 R.**IHFPLATYAPVISAEK**.A

281-304 2750.4100 2749.4027 2749.2840 0.1188 K.**AYHEQLSVAEITNACFEPANQMVK**.C

312-320 1249.5700 1248.5627 1248.5453 0.0174 K.**YMACCLLYR**.G

353-370 1824.9300 1823.9227 1823.9782 -0.0554 K.**VGINYQPPTVVPGGDLAK**.V

374-390 1864.8800 1863.8727 1863.8971 -0.0244 R.**AVCMLSNTTAIAEAWAR**.L

403-422 2330.0200 2329.0127 2329.0110 0.0018 R.**AFVHWYVGEGMEEGEFSEAR**.E

The positions of the tryptic peptides detected within the human α-1A-tubulin amino acid sequence are also shown in bold. The position of K-40, the site of acetylation recognised by a mouse monoclonal antibody is underlined.

1 MRECISIHVG QAGVQIGNAC WELYCLEHGI QPDGQMPSDK TIGGGDDSFN

51 TFFSETGAGK HVPRAVFVDL EPTVIDEVRT GTYR**QLFHPE QLITGKEDAA**

101 **NNYAR**GHYTI GK**EIIDLVLD R**IRKLADQCT GLQGFLVFHS FGGGTGSGFT

151 SLLMERLSVD YGKKSKLEFS IYPAPQVSTA VVEPYNSILT THTTLEHSDC

201 AFMVDNEAIY DICRR**NLDIE RPTYTNLNRL IGQIVSSITA SLRFDGALNV**

251 **DLTEFQTNLV PYPRIHFPLA TYAPVISAEK AYHEQLSVAE ITNACFEPAN**

301 **QMVK**CDPRHG K**YMACCLLYR** GDVVPKDVNA AIATIKTKRT IQFVDWCPTG

351 FK**VGINYQPP TVVPGGDLAK** VQR**AVCMLSN TTAIAEAWAR** LDHKFDLMYA

401 KR**AFVHWYVG EGMEEGEFSE AR**EDMAALEK DYEEVGVDSV EGEGEEEGEE

451 Y

**Tubulin β-2B-chain peptides:**

**St-End Observed Mr(expt) Mr(calc) Delta Sequence**

3-19 1822.9000 1821.8927 1821.9156 -0.0228 R.**EIVHIQAGQCGNQIGAK.**F

47-58 1355.6800 1354.6727 1354.6517 0.0210 R.**INVYYNEAAGNK.**Y

63-77 1615.8500 1614.8427 1614.8287 0.0140 R.**AILVDLEPGTMDSVR.**S

78-103 2798.5400 2797.5327 2797.3361 0.1966 R**.SGPFGQIFRPDNFVFGQSGAGNNWAK.**G

104-121 1958.9300 1957.9227 1957.9745 -0.0518 K**.GHYTEGAELVDSVLDVVR.**K

155-162 1077.5500 1076.5427 1076.5250 0.0177 K**.IREEYPDR.**I

157-174 2141.0000 2139.9927 2139.9969 -0.0042 R**.EEYPDRIMNTFSVMPSPK.**V

217-241 2708.4000 2707.3927 2707.3310 0.0618 K.**LTTPTYGDLNHLVSATMSGVTTCLR.**F

242-251 1130.6300 1129.6227 1129.5880 0.0347 R.**FPGQLNADLR.**K

253-262 1143.6500 1142.6427 1142.6270 0.0157 K.**LAVNMVPFPR.**L

263-276 1620.8600 1619.8527 1619.8283 0.0245 R.**LHFFMPGFAPLTSR.**G

298-306 1065.4700 1064.4627 1064.4201 0.0426 K.**NMMAACDPR.**H

310-318 1053.6200 1052.6127 1052.6019 0.0109 R.**YLTVAAIFR.**G

351-359 1028.5500 1027.5427 1027.5121 0.0307 K.**TAVCDIPPR.**G

381-390 1229.6200 1228.6127 1228.5910 0.0217 R.**ISEQFTAMFR.**R

The positions of the tryptic peptides detected within the human β-2B-tubulin amino acid sequence are also shown in bold:

**1** MR**EIVHIQAG QCGNQIGAK**F WEVISDEHGI DPTGSYHGDS DLQLER**INVY**

**51 YNEAAGNK**YV PR**AILVDLEP GTMDSVRSGP FGQIFRPDNF VFGQSGAGNN**

**101 WAKGHYTEGA ELVDSVLDVV R**KESESCDCL QGFQLTHSLG GGTGSGMGTL

**151** LISK**IREEYP DRIMNTFSVM PSPK**VSDTVV EPYNATLSVH QLVENTDETY

**201** SIDNEALYDI CFRTLK**LTTP TYGDLNHLVS ATMSGVTTCL RFPGQLNADL**

**251 R**K**LAVNMVPF PRLHFFMPGF APLTSR**GSQQ YRALTVPELT QQMFDSK**NMM**

**301 AACDPR**HGR**Y LTVAAIFR**GR MSMKEVDEQM LNVQNKNSSY FVEWIPNNVK

**351** **TAVCDIPPR**G LKMSATFIGN STAIQELFKR **ISEQFTAMFR** RKAFLHWYTG

**401** EGMDEMEFTE AESNMNDLVS EYQQYQDATA DEQGEFEEEE GEDEA

MALDI-TOF mass spectrometry data generated for the initial identification of β-spectrin, and the α3-subunit of the Na^+^,K^+^-ATPase have not been included as this material was further sequence validated using LC MS/MS.

**LC-MS/MS mass spectrometry:**

For the ~270 kDa protein identified as the spectrin β-chain, thirteen tryptic peptides were sequence verified and produced a MOWSE probability score of 345, for which a LC MS/MS MOWSE probability score of greater than 50 was considered a significant match (*P* < 0.05) of tryptic peptides to those present within protein databases, to enable confident protein identification. The tryptic peptides identified constituted ~7 % of the protein sequence coverage:

**St-End Observed Mr(expt) Mr(calc) Delta Sequence**

75-83 555.2958 1108.5770 1108.5764 0.0006 R.**ITDLYTDLR**.D

250-264 574.6010 1720.7812 1720.8156 -0.0344 K.**LLDPEDISVDHPDEK**.S

724-736 779.4109 1556.8072 1556.7947 0.0126 R.**EQWANLEQLSAIR**.K

843-853 632.3511 1262.6876 1262.6870 0.0006 K.**QALQDTLALYK**.M

1018-1036 717.6721 2149.9945 2150.0603 -0.0658 K.**EAEKLESEHPDQAQAILSR**.L

1162-1176 611.6397 1831.8973 1831.9329 -0.0356 R.**QNLLSQSHAYQQFLR**.D

1467-1477 703.8558 1405.6970 1405.6911 0.0059 K.**FMELLEPLNER**.K

1706-1717 751.8364 1501.6582 1501.7049 -0.0466 R.**EVDDLEQWIAER**.E

1798-1809 687.3835 1372.7524 1372.7350 0.0174 R.**TQILAASYELHK**.F

1845-1861 667.3230 1998.9472 1998.9582 -0.0110 R.**MHTTFEHDIQALGTQVR**.Q

2079-2088 593.8505 1185.6864 1185.6969 -0.0104 R.**LTTLELLEVR**.R

2178-2191 684.3809 1366.7472 1366.7568 -0.0096 K.**TALPAQSAATLPAR**.T

2279-2290 706.3459 1410.6772 1410.6779 -0.0007 R.**LNDGNEYLFQAK**.D

The positions of the tryptic peptides detected within the human β-spectrin amino acid sequence are also shown in bold:

**1** MTTTVATDYD NIEIQQQYSD VNNRWDVDDW DNENSSARLF ERSRIKALAD

**51** EREAVQKKTF TKWVNSHLAR VSCR**ITDLYT DLR**DGRMLIK LLEVLSGERL

**101** PKPTKGRMRI HCLENVDKAL QFLKEQRVHL ENMGSHDIVD GNHRLTLGLI

**151** WTIILRFQIQ DISVETEDNK EKKSAKDALL LWCQMKTAGY PNVNIHNFTT

**201** SWRDGMAFNA LIHKHRPDLI DFDKLKKSNA HYNLQNAFNL AEQHLGLTK**L**

**251 LDPEDISVDH PDEK**SIITYV VTYYHYFSKM KALAVEGKRI GKVLDNAIET

**301** EKMIEKYESL ASDLLEWIEQ TIIILNNRKF ANSLVGVQQQ LQAFNTYRTV

**351** EKPPKFTEKG NLEVLLFTIQ SKMRANNQKV YMPREGKLIS DINKAWERLE

**401** KAEHERELAL RNELIRQEKL EQLARRFDRK AAMRETWLSE NQRLVSQDNF

**451** GFDLPAVEAA TKKHEAIETD IAAYEERVQA VVAVARELEA ENYHDIKRIT

**501** ARKDNVIRLW EYLLELLRAR RQRLEMNLGL QKIFQEMLYI MDWMDEMKVL

**551** VLSQDYGKHL LGVEDLLQKH TLVEADIGIQ AERVRGVNAS AQKFATDGEG

**601** YKPCDPQVIR DRVAHMEFCY QELCQLAAER RARLEESRRL WKFFWEMAEE

**651** EGWIREKEKI LSSDDYGKDL TSVMRLLSKH RAFEDEMSGR SGHFEQAIKE

**701** GEDMIAEEHF GSEKIRERII YIR**EQWANLE QLSAIR**KKRL EEASLLHQFQ

**751** ADADDIDAWM LDILKIVSSS DVGHDEYSTQ SLVKKHKDVA EEIANYRPTL

**801** DTLHEQASAL PQEHAESPDV RGRLSGIEER YKEVAELTRL RK**QALQDTLA**

**851 LYK**MFSEADA CELWIDEKEQ WLNNMQIPEK LEDLEVIQHR FESLEPEMNN

**901** QASRVAVVNQ IARQLMHSGH PSEKEIKAQQ DKLNTRWSQF RELVDRKKDA

**951** LLSALSIQNY HLECNETKSW IREKTKVIES TQDLGNDLAG VMALQRKLTG

**1001** MERDLVAIEA KLSDLQK**EAE KLESEHPDQA QAILSR**LAEI SDVWEEMKTT

**1051** LKNREASLGE ASKLQQFLRD LDDFQSWLSR TQTAIASEDM PNTLTEAEKL

**1101** LTQHENIKNE IDNYEEDYQK MRDMGEMVTQ GQTDAQYMFL RQRLQALDTG

**1151** WNELHKMWEN R**QNLLSQSHA YQQFLR**DTKQ AEAFLNNQEY VLAHTEMPTT

**1201** LEGAEAAIKK QEDFMTTMDA NEEKINAVVE TGRRLVSDGN INSDRIQEKV

**1251** DSIDDRHRKN RETASELLMR LKDNRDLQKF LQDCQELSLW INEKMLTAQD

**1301** MSYDEARNLH SKWLKHQAFM AELASNKEWL DKIEKEGMQL ISEKPETEAV

**1351** VKEKLTGLHK MWEVLESTTQ TKAQRLFDAN KAELFTQSCA DLDKWLHGLE

**1401** SQIQSDDYGK HLTSVNILLK KQQMLENQME VRKKEIEELQ SQAQALSQEG

**1451** KSTDEVDSKR LTVQTK**FMEL LEPLNER**KHN LLASKEIHQF NRDVEDEILW

**1501** VGERMPLATS TDHGHNLQTV QLLIKKNQTL QKEIQGHQPR IDDIFERSQN

**1551** IVTDSSSLSA EAIRQRLADL KQLWGLLIEE TEKRHRRLEE AHRAQQYYFD

**1601** AAEAEAWMSE QELYMMSEEK AKDEQSAVSM LKKHQILEQA VEDYAETVHQ

**1651** LSKTSRALVA DSHPESERIS MRQSKVDKLY AGLKDLAEER RGKLDERHRL

**1701** FQLNR**EVDDL EQWIAER**EVV AGSHELGQDY EHVTMLQERF REFARDTGNI

**1751** GQERVDTVNH LADELINSGH SDAATIAEWK DGLNEAWADL LELIDTR**TQI**

**1801 LAASYELHK**F YHDAKEIFGR IQDKHKKLPE ELGRDQNTVE TLQR**MHTTFE**

**1851 HDIQALGTQV R**QLQEDAARL QAAYAGDKAD DIQKRENEVL EAWKSLLDAC

**1901** ESRRVRLVDT GDKFRFFSMV RDLMLWMEDV IRQIEAQEKP RDVSSVELLM

**1951** NNHQGIKAEI DARNDSFTTC IELGKSLLAR KHYASEEIKE KLLQLTEKRK

**2001** EMIDKWEDRW EWLRLILEVH QFSRDASVAE AWLLGQEPYL SSREIGQSVD

**2051** EVEKLIKRHE AFEKSAATWD ERFSALER**LT TLELLEVR**RQ QEEEERKRRP

**2101** PSPEPSTKVS EEAESQQQWD TSKGEQVSQN GLPAEQGSPR MAETVDTSEM

**2151** VNGATEQRTS SKESSPIPSP TSDRKAK**TAL PAQSAATLPA R**TQETPSAQM

**2201** EGFLNRKHEW EAHNKKASSR SWHNVYCVIN NQEMGFYKDA KTAASGIPYH

**2251** SEVPVSLKEA VCEVALDYKK KKHVFKLR**LN DGNEYLFQAK** DDEEMNTWIQ

**2301** AISSAISSDK HEVSASTQST PASSRAQTLP TSVVTITSES SPGKREKDKE

**2351** KDKEKRFSLF GKKK

For the ~112 kDa protein identified as the catalytic α3-subunit of human Na^+^,K^+^-ATPase, fourteen tryptic peptides were sequence verified and produced a MOWSE probability score of 724. A MOWSE probability score of > 50 was considered a significant match of tryptic peptides (*P* < 0.05) to those present within protein databases, to enable confident protein identification. The fourteen tryptic peptides identified constituted ~19 % of the protein sequence coverage:

**St-End Observed Mr(expt) Mr(calc) Delta Sequence**

65-81 910.4627 1818.9108 1818.9152 -0.0044 R.**DGPNALTPPPTTPEWVK**.F

153-163 634.8702 1267.7258 1267.7071 0.0188 K.**NMVPQQALVIR**.E

203-217 810.3666 1618.7186 1618.7435 -0.0248 K.**VDNSSLTGESEPQTR**.S

260-271 579.8227 1157.6308 1157.6656 -0.0347 R.**IATLASGLEVGK**.T

350-367 962.9396 1923.8646 1923.9095 -0.0449 K.**NLEAVETLGSTSTICSDK**.T

404-420 636.9733 1907.8981 1907.9424 -0.0444 K.**SSHTWVALSHIAGLCNR**.A

425-434 520.7778 1039.5410 1039.5662 -0.0252 K.**GGQDNIPVLK**.R

436-448 638.3085 1274.6024 1274.6354 -0.0329 R.**DVAGDASESALLK**.C

603-615 461.2423 1380.7051 1380.7435 -0.0385 K.**VIMVTGDHPITAK**.A

620-637 915.4435 1828.8724 1828.9167 -0.0442 K.**GVGIISEGNETVEDIAAR**.L

638-648 618.8433 1235.6720 1235.6986 -0.0266 R.**LNIPVSQVNPR**.D

698-716 906.4695 1810.9244 1810.9425 -0.0181 R.**QGAIVAVTGDGVNDSPALK**.K

718-733 753.8572 1505.6998 1505.7396 -0.0397 K.**ADIGVAMGIAGSDVSK**.Q

757-764 495.7957 989.5768 989.5909 -0.0141 R.**LIFDNLKK**.S

The positions of the tryptic peptides detected within the human α3-subunit of human Na^+^,K^+^-ATPase amino acid sequence are also shown in bold:

**1** MGDKKDDKDS PKKNKGKERR DLDDLKKEVA MTEHKMSVEE VCRKYNTDCV

**51** QGLTHSKAQE ILAR**DGPNAL TPPPTTPEWV K**FCRQLFGGF SILLWIGAIL

**101** CFLAYGIQAG TEDDPSGDNL YLGIVLAAVV IITGCFSYYQ EAKSSKIMES

**151** FK**NMVPQQAL VIR**EGEKMQV NAEEVVVGDL VEIKGGDRVP ADLRIISAHG

**201** CK**VDNSSLTG ESEPQTR**SPD CTHDNPLETR NITFFSTNCV EGTARGVVVA

**251** TGDRTVMGR**I ATLASGLEVG K**TPIAIEIEH FIQLITGVAV FLGVSFFILS

**301** LILGYTWLEA VIFLIGIIVA NVPEGLLATV TVCLTVTAKR MARKNCLVK**N**

**351 LEAVETLGST STICSDK**TGT LTQNRMTVAH MWFDNQIHEA DTTEDQSGTS

**401** FDK**SSHTWVA LSHIAGLCNR** AVFK**GGQDNI PVLK**R**DVAGD ASESALLK**CI

**451** ELSSGSVKLM RERNKKVAEI PFNSTNKYQL SIHETEDPND NRYLLVMKGA

**501** PERILDRCST ILLQGKEQPL DEEMKEAFQN AYLELGGLGE RVLGFCHYYL

**551** PEEQYPQGFA FDCDDVNFTT DNLCFVPLMS MIGPPRAAVP DAVGKCRSAG

**601** IK**VIMVTGDH PITAK**AIAK**G VGIISEGNET VEDIAARLNI PVSQVNPR**DA

**651** KACVIHGTDL KDFTSEQIDE ILQNHTEIVF ARTSPQQKLI IVEGCQR**QGA**

**701 IVAVTGDGVN DSPALK**K**ADI GVAMGIAGSD VSK**QAADMIL LDDNFASIVT

**751** GVEEGR**LIFD NLKK**SIAYTL TSNIPEITPF LLFIMANIPL PLGTITILCI

**801** DLGTDMVPAI SLAYEAAESD IMKRQPRNPR TDKLVNERLI SMAYGQIGMI

**851** QALGGFFSYF VILAENGFLP GNLVGIRLNW DDRTVNDLED SYGQQWTYEQ

**901** RKVVEFTCHT AFFVSIVVVQ WADLIICKTR RNSVFQQGMK NKILIFGLFE

**951** ETALAAFLSY CPGMDVALRM YPLKPSWWFC AFPYSFLIFV YDEIRKLILR

**1001** RNPGGSVEKE TYY
